# Clinical efficacy and safety of rimegepant in the treatment of migraine: a meta-analysis of randomized controlled trials

**DOI:** 10.3389/fneur.2023.1205778

**Published:** 2023-06-20

**Authors:** Qinghui Wang, Shuangmei Wang, Yi Zhu, Fei Lin

**Affiliations:** ^1^Department of Pharmacy, Chengdu Jinniu District People's Hospital, Chengdu, Sichuan, China; ^2^Department of Pharmacy, The First Affiliated Hospital of Chengdu Medical College, Chengdu, Sichuan, China

**Keywords:** migraine, rimegepant, meta-analysis, efficacy, safety

## Abstract

**Background:**

This study aims to evaluate the clinical efficacy and safety of rimegepant for the treatment of migraine in adult patients using a meta-analysis.

**Methods:**

The PubMed, EMBASE, and Cochrane Library were searched up to March 2022. Only randomized controlled trials (RCTs) that evaluated migraine and other comparator treatments in adult patients were included. The clinical response at the post-treatment evaluation, including acute pain free and relief effect, whereas the secondary outcomes were the risk of adverse events (AEs).

**Results:**

A total of 4 RCTs involving 4,230 patients with episodic migraine were included. Outcome indicators for the number of pain free and relief patients at 2 h, 2–24 h, 2–48 h post-dose showed that rimegepant had better effects relative to the placebo [free at 2 h: OR = 1.84, 95% CI (1.55, 2.18), *P* < 0.00001; relief at 2 h: OR = 1.80, 95% CI (1.59, 2.04), *P* < 0.00001]. And there was no significant difference between the occurrence of adverse events in the experimental and control groups [OR = 1.29, 95% CI (0.99, 1.67), *P* = 0.06].

**Conclusion:**

Rimegepant has better therapeutic effects compared to placebo and no significant difference in adverse events.

## Introduction

Migraine is one of the most common causes of severe headaches in the world (1). People who suffer from frequent migraines may lose more than half their lives because of migraines (2). Migraine has been listed as the second most common disabling disorder worldwide by the Global Burden of Disease Study (GBD) lately and first among young women (3–6). Currently, there is no cure for migraine, only symptom control. For the treatment of acute attacks, over-the-counter medications such as ibuprofen are available, mostly non-specific, and among the specific prescription drugs, triptans are the more common choice (7). Introduced in the 1990s, triptans are targeted but not effective for everyone because of their vasoconstrictive effect and are not indicated for people with cardiovascular disease or associated risk factors (8, 9). Calcitonin gene-related peptide (CGRP) is a neurotransmitter with vasodilatory effects. CGRP and its receptors are expressed in neurological regions associated with migraine pathophysiology. During migraine attacks, the level of CGRP release is significantly elevated and is considered to be an important trigger of migraine (10). Furthermore, the procedure does not cause any other vasoconstriction. Thus, inhibition of the CGRP signaling pathway is a novel mechanism of action for the acute treatment of migraine, and the CGRP receptor is now a popular target for migraine drug development (11, 12).

There are several monoclonal migraine drugs targeting the CGRP receptor that have been marketed worldwide (10). In 2018, the U.S. Food and Drug Administration (FDA) approved three migraine prevention drugs targeting CGRP itself or its receptor, all injectable. Rimegepant is an oral CGRP receptor antagonist with a more convenient dosing regimen (12).

Recently, the FDA expanded the indication for rimegepant to be used for the prophylactic treatment of migraine in adults and approved it as the first CGRP receptor antagonist to be approved as an efficacious orally disintegrating tablet for the acute treatment of migraine in adults with or without aura. The drug is currently the only migraine medication that both treats acute migraine attacks and helps prevent future attacks. But some scholars believe that rimegepant is the new garment of the emperor because of the lack of study patients (13).

Thus, we will increase the number of studies based on the existing studies and conduct a meta-analysis of the efficacy of rimegepant to obtain a more realistic clinical efficacy of rimegepant, while meanwhile assessing the effect of the treatment dose on migraine control and adverse effects. Our study summarizes efficacy and safety studies from multiple randomized controlled trails, offsetting the lag caused by the number of patients included.

## Methods

### Study search and selection

We followed the PRISMA guidelines throughout the formation process of our study. We searched PubMed, Embase, and the Cochrane Library. The following search terms were used: “Rimegepant” (Supplementary Concept) OR BMS-927711 Studies were included if they met the following criteria: (a) the RCT; (b) patients had a history of migraine for at least 1 year before the age of 50, 2–8 moderate or severe migraine attacks per month, and <15 days per month for the previous 3 months; (c) the intervention of rimegepant and comparison with other medications to treat migraines; and (d) the outcome of efficacy, including pain relief 2 h after administration and AEs. All languages of publication could be included. However, studies were excluded if they met the following criteria: (a) *in vitro* studies; (b) pharmacokinetic-pharmacodynamic assessment; (c) review and abstract; and (d) phase I trials. Two reviewers (Qinghui Wang and Fei Lin) searched and examined publications independently to avoid bias. A third reviewer (Yi Zhu) resolved and decided on any disagreements. The following data were extracted from all the included studies: authorship, year of publication, study design, study duration, study site, study population, participants and comparators, clinical outcomes, and risk of adverse events (AEs). The modified intent-to-treat (MITT) population included all ITT patients with a confirmed diagnosis and conditions that met the study protocol criteria. The clinically evaluable (CE) population included patients from the MITT population who had a qualifying symptom as per the criteria for trial entry, received a trial drug, did not receive any medication not assigned within the trial that could confound interpretation of the results, and had an assessment of outcome during the protocol-defined window. Our institute did not require ethical approval for systematic reviews and meta-analyses.

### Outcome measurement

We observed several indicators of pain free and pain relief to better describe the efficacy. Including migraine pain free at 2 h, without the use of rescue medication; the second set of endpoints included migraine relief at 2 h without the use of rescue medication. Then we, respectively, assessed sustained pain free and relief at 2–24 h and sustained pain free and relief from the most bothersome symptom (MBS) at 2–48 h. We screened the articles and assessed the results as per the guidelines (14). Treatment-emergent adverse events (TEAEs) were recorded, regardless of causality.

### Data analysis

The Review Manager 5.2 software was used to create the risk of bias plot in individual studies. And the Cochrane risk-of-bias tool was used to assess the quality of the included RCTs and their associated risk of bias. Statistical analyses were performed with the fixed-effects model. Pooled odds ratios (ORs) and 95% confidence intervals (CIs) were calculated for outcome analyses. The degree of heterogeneity was evaluated with the Chi-squared test. The proportion of statistical heterogeneity was assessed using the *I*^2^ measure. Heterogeneity was considered significant when *P* < 0.10 or *I*^2^ > 50%. The random-effect model was used when the data were significantly heterogeneous, and the fixed-effect model was used when the data were homogeneous. First, analyze the causes of heterogeneity. Subgroup analysis, sensitive analysis, Breslow-Day, and regression approximation can be used for factors that may produce clinical and statistical heterogeneity. If the heterogeneity cannot be eliminated after excluding interference factors, a random effect model (REM) can be selected for pooled data analysis. Combined odds ratios (OR) and 95% confidence intervals (CIs) were calculated for outcome analyses.

## Results

### Search and study characteristics

**Figure 1:**
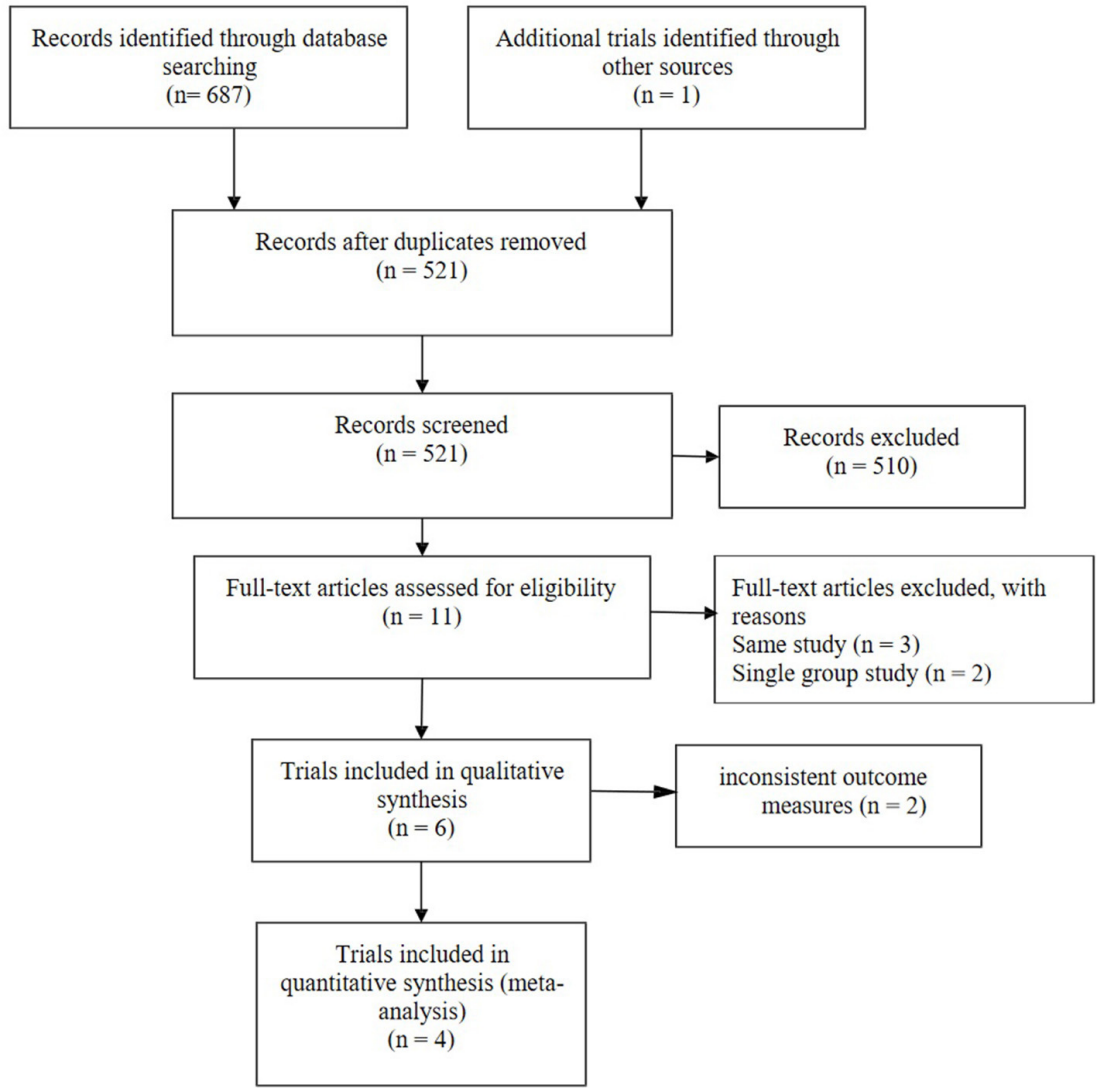
The study search, selection, and inclusion process.

A total of 688 research articles and abstracts from Pubmed, Embase, and the Cochrane Library were identified. One hundred and eight studies were removed due to duplicates. After removing duplicates and uncorrelated titles, eleven of these articles were directly related to the topic of interest. There are seven full-text articles excluded, with reasons as follows: same study (*n* = 3), single group study (*n* = 2), and inconsistent outcome measures (*n* = 2). Finally, four RCTs containing 4,230 patients were included in our meta-analysis. The specific process and included study characteristics are shown in Figure 1.

### Basic characteristics of the included studies and risk of bias evaluation result

We summarized the basic characteristics of included studies in the order of publication years, and them are listed in Table 1.

**Table 1:** Basic characteristics of included studies.

**Included studies**	**Research**	**Follow-up time**	**Number of cases (intervening measure)**	**Mean age (mean value ±SD/median)**	**Sex (F/M)**
Marcus et al. (15)	NCT01430442	12 weeks	85 (BMS 927711 10 mg)	41.1 ± 10.36	67/79
			68 (BMS 927711 25 mg)	36.5 ± 11.92	61/90
			91 (BMS 927711 75 mg)	38.5 ± 11.87	81/89
			90 (BMS 927711 150 mg)	39.2 ± 11.26	63/70
			121 (BMS 927711 300 mg)	41.9 ± 11.46	101/84
			92 (BMS 927711 600 mg)	39.3 ± 13.01	76/83
			109 (Sumatriptan)	40.6 ± 10.47	91/84
			229 (Placebo)	37.9 ± 11.36	196/86
NCT03235479. 2018	NCT03235479	45 days	543 (BHV-3000 75 mg)	41.945 ± 12.33	464/79
			541 (Placebo)	41.326 ± 12.14	463/78
Croop et al. (16)	NCT03461757	52 days	669 (Rimegepant 75 mg)	40.3 ± 12.1	568/101
			682 (Placebo)	40.0 ± 11.9	579/103
Lipton et al. (17)	NCT03237845	45 days	537 (Rimegepant 75 mg)	40.2 ± 11.9	479/58
			535 (Placebo)	40.9 ± 12.1	472/63

**Figure 2:**
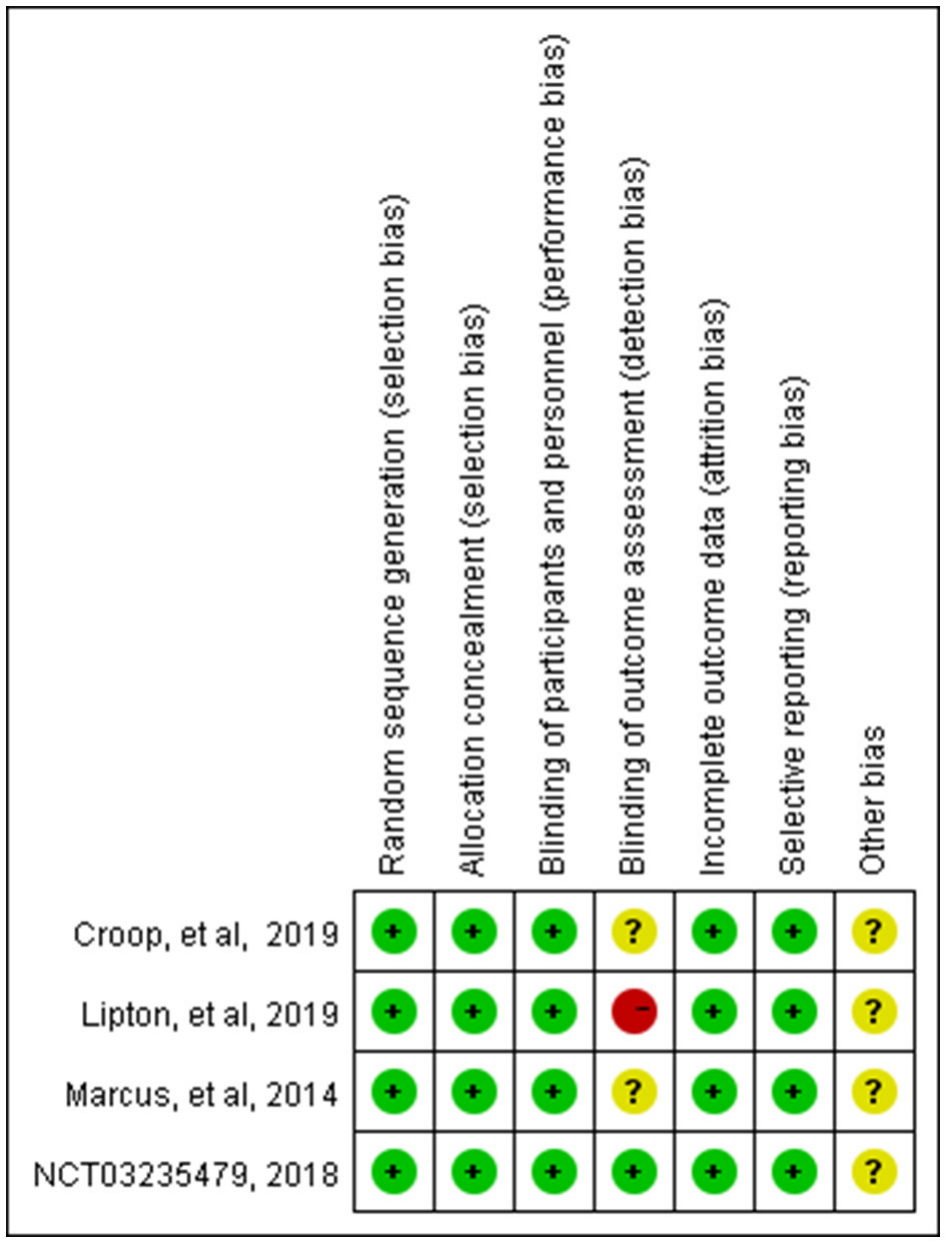
Bias evaluate in the meta-analysis.

**Figure 3:**
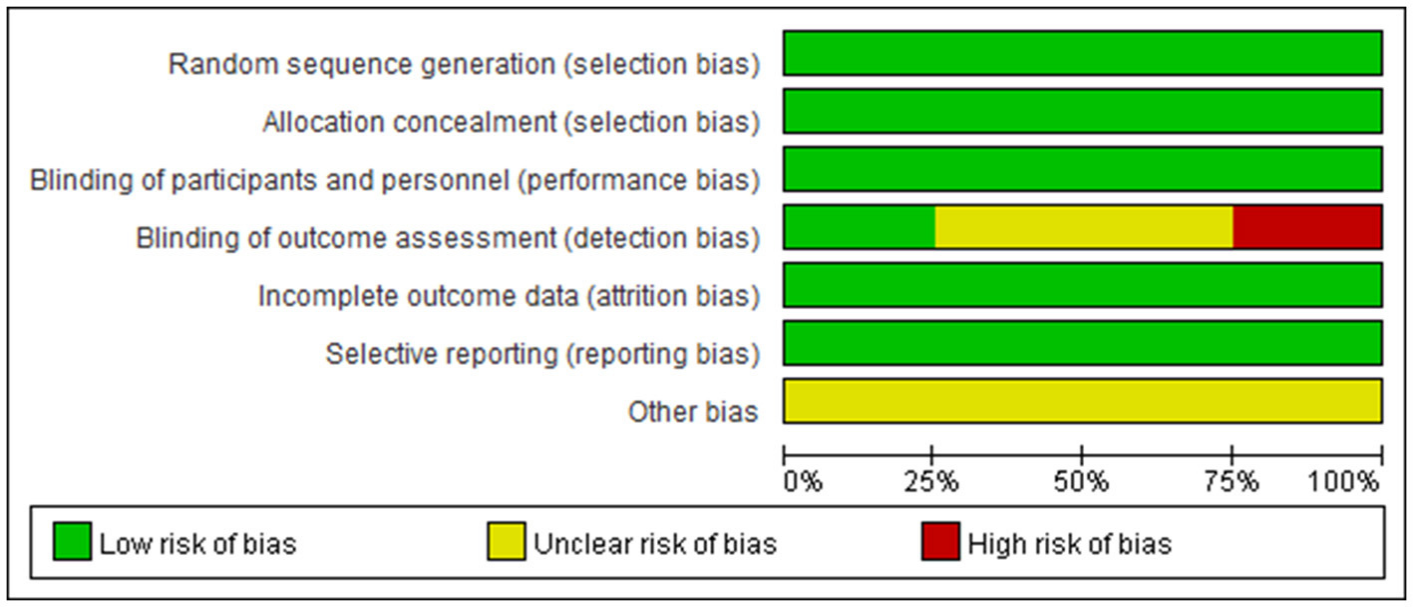
Bias analysis in the meta-analysis.

These studies were all registered with ClinicalTrials.gov, and their numbers were listed in the table. The basic characteristics showed that the average age of the patients in the four studies showed no distinct difference; they were all around 40 years old, with more women than men. The Cochrane risk-of-bias tool was used to assess the risk of bias in our study, and the detailed results are shown in Figures 2, 3.

### Clinical efficacy of rimegepant in the treatment of migraine

**Figure 4:**
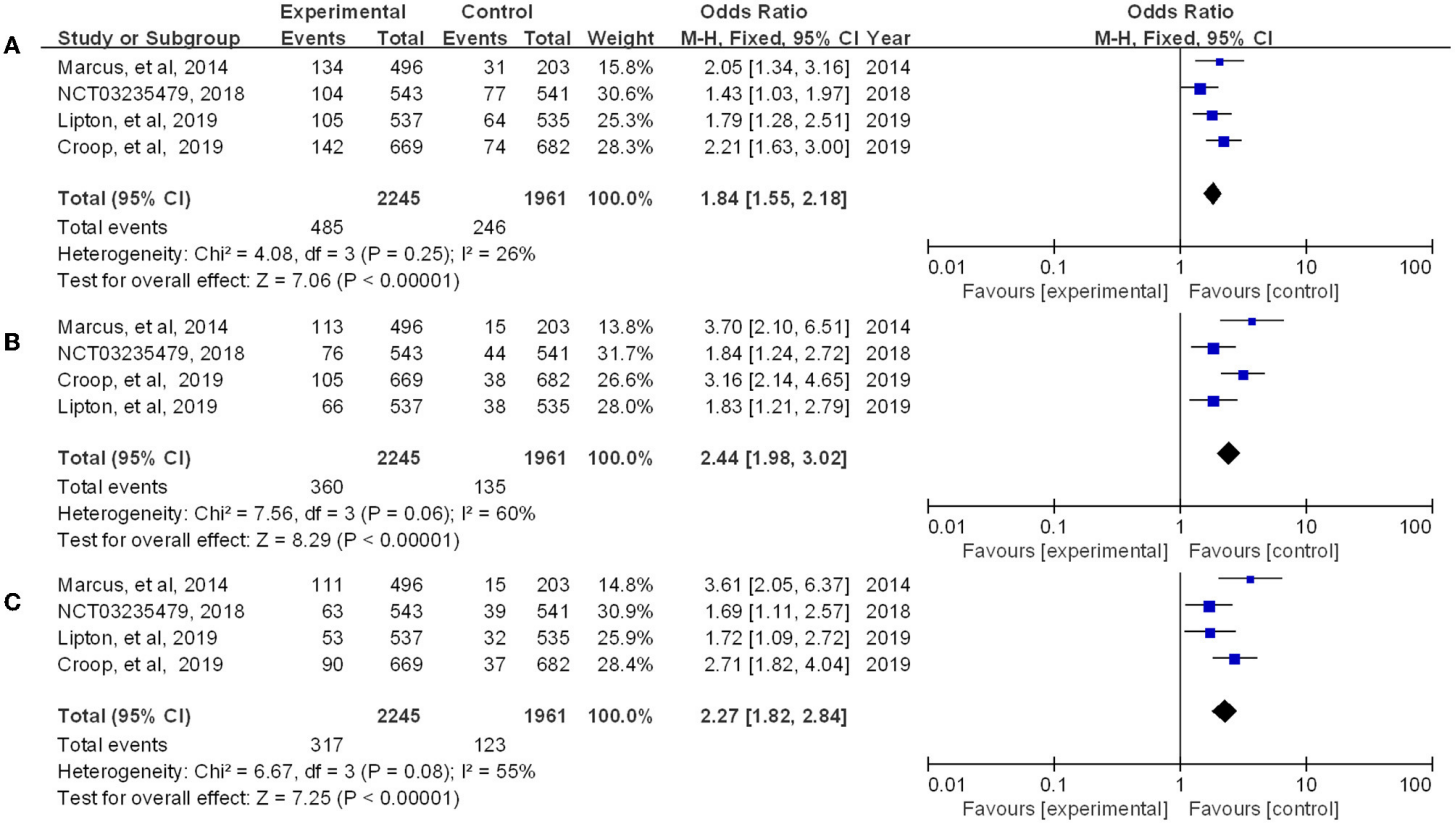
Forest of patients with pain freedom (**A:** 2 h post-dose; **B**: 2–24 h post-dose; **C**: 2–48 h post-dose).

**Figure 5:**
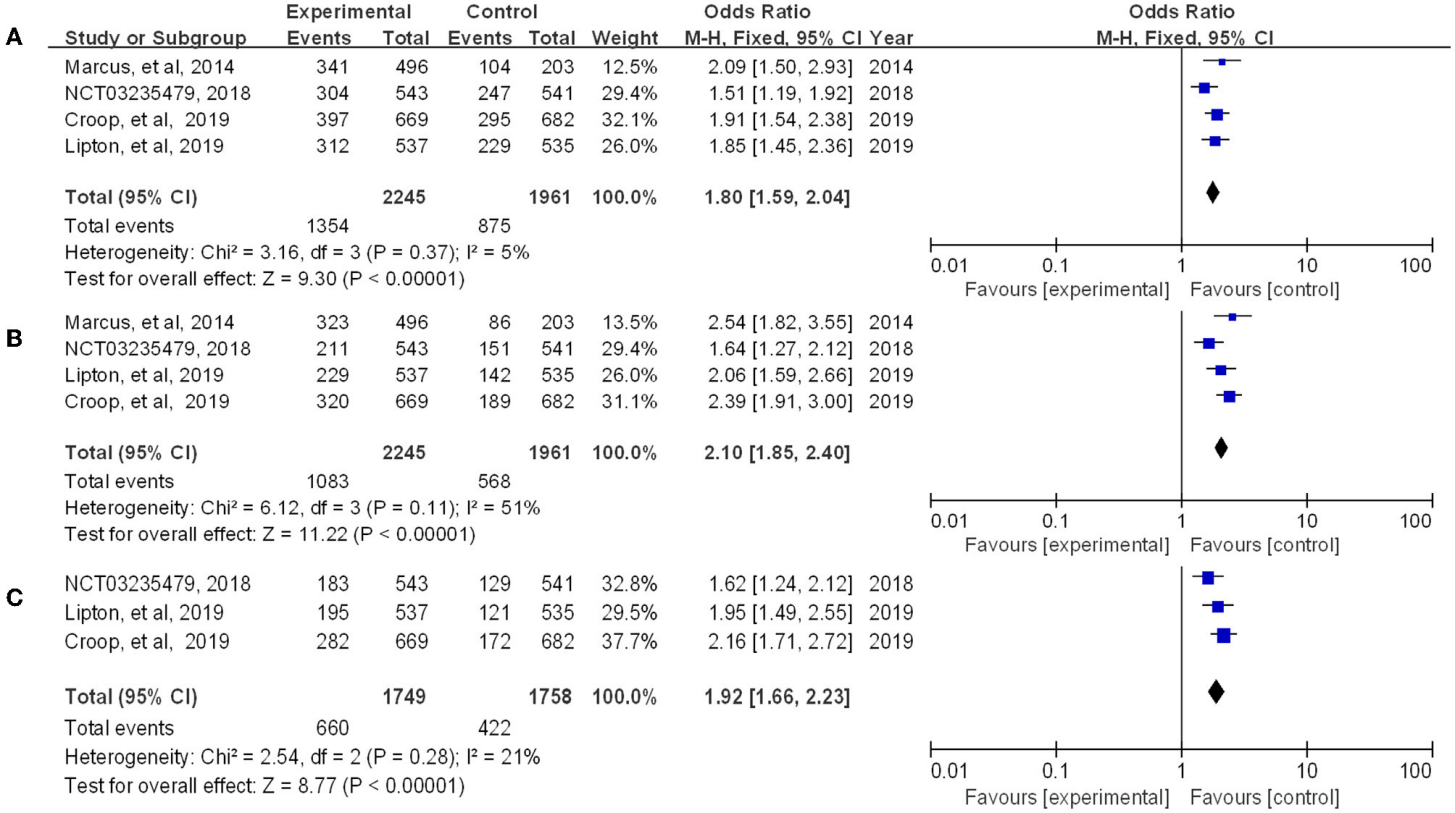
Forest of patients with pain relief (**A**: 2 h post-dose; **B**: 2–24 h post-dose; **C**: 2–48 h post-dose).

Rimegepant, one of the CGRP small molecule antagonists, has been extensively studied in terms of *in vivo* absorption, and drug concentration (18). Multiple indicators showed that rimegepant was more effective than placebo in our study. The meta-analysis found that rimegepant outperformed placebo in terms of pain freedom at 2 h post-dose [OR = 1.84, 95% CI (1.55, 2.18)] and pain relief at 2 h post-dose [OR = 1.80, 95% CI (1.59, 2.04)]. Moreover, rimegepant had better effects than placebo in patients who were pain free at 2–24 h post-dose [OR = 2.44, 95% CI (1.98, 3.02)], pain relief patients at 2–24 h post-dose [OR = 2.1, 95% CI (1.85, 2.40)], pain free at 2–48 h post-dose [OR = 2.27, 95% CI (1.82, 2.84)], and pain relief at 2–48 h post-dose [OR = 1.92, 95% CI (1.66, 2.23)]. The detailed results of patients with pain freedom at 2 hours, 2–24 hours, and 2–48 hours post-dose are listed in Figure 4, and pain relief after these time are listed in Figure 5.

### Clinical safety of rimegepant in the treatment of migraine

**Figure 6:**
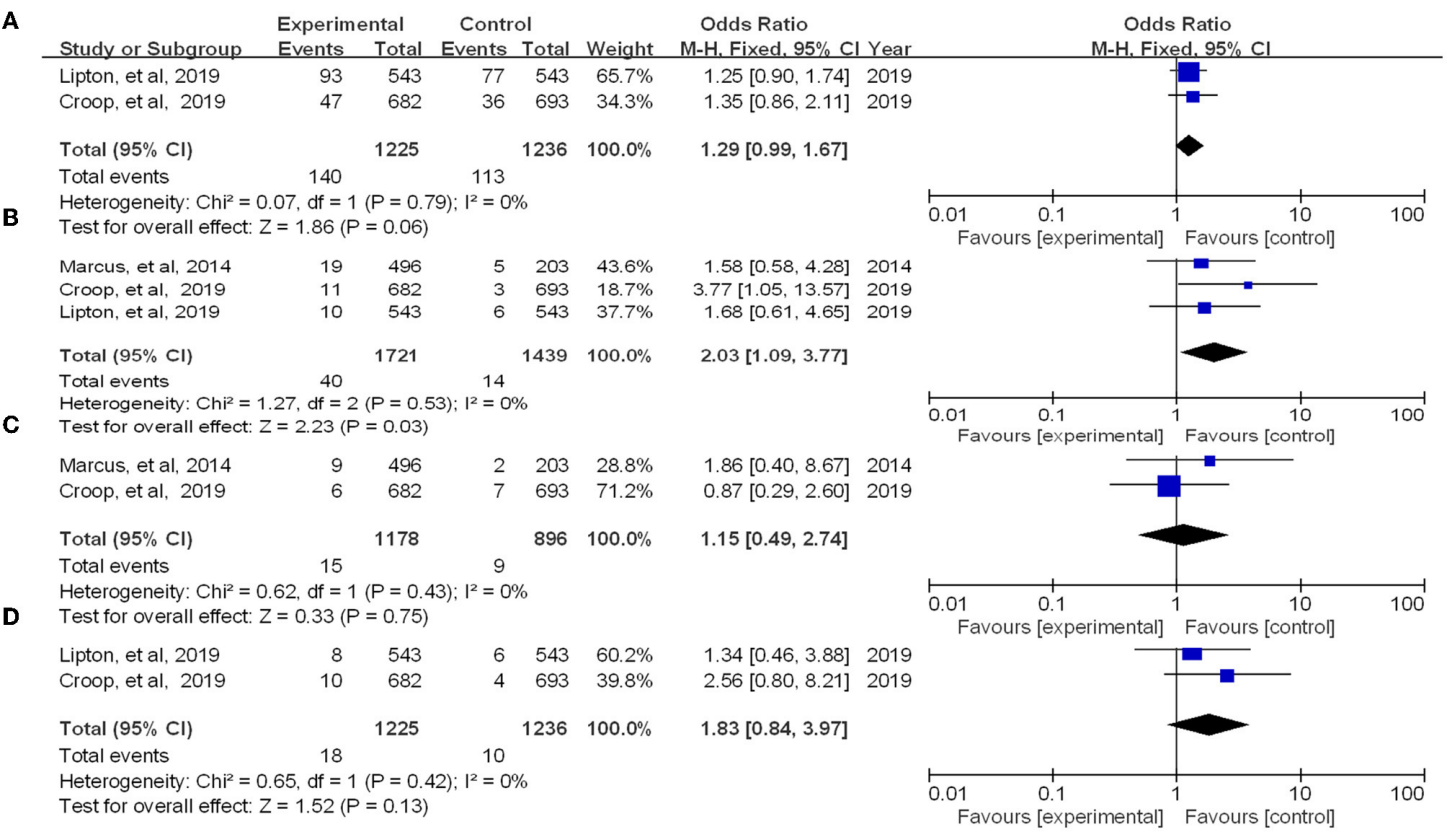
Forest of patients with adverse event (**A**: all adverse event; **B**: nausea; **C**: dizziness; **D**: urinary tract infection).

Four RCTs reporting treatment-associated adverse events associated with therapy. We summarized the overall number of adverse events in the experimental group vs. the placebo group and performed a meta-analysis of the experimental results. The results showed that there was no significant difference between the occurrence of adverse reactions in the experimental and control groups [OR = 1.29, 95% CI (0.99, 1.67), *P* = 0.06] (Figure 6). Then we tested for the most common side effects found in previous studies, such as nausea, dizziness, and urinary tract infections. Among them, the experimental group showed significant differences in nausea compared with the placebo group (*P* = 0.03) but no significant difference in dizziness or urinary tract infection. As side effects, reported adverse events include vomiting, diarrhea, paresthesia, dysgeusia, chest discomfort, myalgia, etc. (15).

## Discussion

In this study, we included a total of 4 RCT studies, including 4,230 patients. In February 2020, rimegepant received its first global approval in the USA for the acute treatment of migraine (aura) in adults (19). So we assessed the effect of rimegepant on migraine control and safety in clinical use. Baseline data indicate that the proportion of female and middle-aged patients is large, consistent with the data reported by GBD (6). Previous pharmacotherapy for acute migraine focused on short-term treatment, focusing on pain freedom 2 h post-dose (18). However, for patients with severe migraine, the evaluation index of 2-h remission inevitably leads to a deviation in the results. Therefore, we included pain free and relief as the primary outcome and increased the time from the previous 2 h to 2–48 h.

Our results indicate that in terms of efficacy, both for 2 h, 2–24 h, 2–48 h post-dose symptom-free and relief, the efficacy of the rimegepant is significantly different from that of the placebo. One study (15) has shown that compared to triptans, the therapeutics effect of rimegepant is limited, but it is still an optional medicine for taboo patients, such as patients with cardiovascular patients. For the migraine control effect achieved by long-term drug treatment, Johnston et al. (20) find monthly migraine days in rimegepant change from baseline is reduced by 3.31 days [95% CI (3.75, −2.87)] in 4 – 14 MMD QOD 1 + PRN. According to Croop et al. (21), monthly migraine days of rimegepant reduce by 4.3 days [95% CI (4.8, 3.9)] and of placebo, by 3.5 days (−4.0, 3.0). In addition, rimegepant reduced lost productivity time due to migraine by 50%. Rimegepant acts as a CGRP antagonist for systemic administration and has shown efficacy in pain freedom and relief, migraine symptom release, and lifestyle recovery (22). We further find out the role of the neuropeptide CGRP in the pathophysiology of migraine, CGRP is the most potent vasodilating peptide known (23), but it has a limited ability to cross the blood-brain barrier (BBB) (24). In the meninges, CGRP may promote neurogenic inflammation by triggering the release of neuronal sensitizers by mast cells, which subsequently leads to increased dural vasodilation (25). Regulation of meningeal neuronal activity can trigger a feedback loop which eventually leads to peripheral sensitization of nocicepters (26).

In terms of adverse reactions, the most common adverse effects of rimegepant were vomiting, dizziness, and urinary tract infections. We find no significant difference between the occurrence of adverse reactions in the rimegepant group and the control groups. For several adverse events commonly reported, nausea was significantly different from the control group; dizziness and urinary tract infection were not different compared with the control group, and previous research did not show any effect on liver function.

Our advantage is that we simultaneously evaluated the efficacy of pain free and pain relief after rimegepant 2 h, 2–24 h, and 2–48 h post-dose, reducing the possible errors of assessing pain free after 2 h alone. Meanwhile, we evaluated the role of long-term time-based quantitative use for migraine prophylactics, and several studies showed that long-term timed use of rimegepant contributed to the number of fewer migraine attacks per month.

Of course, there are also some limitations in this paper. Our study was not the first meta-analysis, and we didn't register, but the number of patients and studies has increased significantly in comparison to previous research. This study did not contain other anti-CGRP drugs or compare the efficacy of rimegepant with other drugs. Studies have shown that anti-CGRP monoclonal antibodies are also effective, safe, and well-tolerated drugs (27). When developing medicines, several aspects should be taken into account, as there are medicines with different mechanisms.

Migraine is highly associated with disability (28). It is currently believed that patients with more frequent and severe migraines will benefit from prophylactic treatment. Modern medical treatment should consider not only the patient's symptoms, diagnosis, and comorbidities but also the patient's expectations (29). Preclinical data and clinical models of migraine are the basis for developing therapeutic agents (30). Our current systematic assessment and meta-analysis primarily assessed the efficacy and safety of randomized controlled trials in the treatment of migraine patients, providing evidence to support for clinical treatment.

## Conclusion

In summary, our study confirms that rimegepant has better therapeutic effects compared to placebo and no significant difference in adverse events.

## Data availability statement

The original contributions presented in the study are included in the article/supplementary material, further inquiries can be directed to the corresponding author.

## Author contributions

QW and FL conceived and designed the study. QW, SW, YZ, and FL analyzed the data and wrote the manuscript. All authors reviewed and approved the final version of the manuscript.

## References

[1] Gawde P Shah H Patel H Bharathi KS Patel N Sethi Y . Revisiting migraine: the evolving pathophysiology and the expanding management armamentarium. Cureus. (2023) 15:e34553. 10.7759/cureus.34553 36879707 PMC9985459

[2] Borkum JM. Migraine triggers and oxidative stress: a narrative review and synthesis. Headache. (2016) 56:12–35. 10.1111/head.12725 26639834

[3] GBD 2015 Disease and Injury Incidence and Prevalence Collaborators. Global, regional, national incidence prevalence, and years lived with disability for 310 diseases and injuries, 1990-2015: a systematic analysis for the Global Burden of Disease Study 2015. Lancet. (2016) 388:1545–602. 10.1016/S0140-6736(16)31678-6 27733282 PMC5055577

[4] GBD 2016 Disease and Injury Incidence and Prevalence Collaborators. Global, regional, national incidence. prevalence, and years lived with disability for 328 diseases and injuries for 195 countries, 1990-2016: a systematic analysis for the Global Burden of Disease Study 2016. Lancet. (2017) 390:1211–59. 10.1016/S0140-6736(17)32154-2 28919117 PMC5605509

[5] GBD 2017 Disease and Injury Incidence and Prevalence Collaborators. Global, regional, national incidence. prevalence, and years lived with disability for 354 diseases and injuries for 195 countries and territories, 1990-2017: a systematic analysis for the Global Burden of Disease Study 2017. Lancet. (2018) 392:1789–858. 10.1016/S0140-6736(18)32279-7 30496104 PMC6227754

[6] Steiner TJ Stovner LJ Jensen R Uluduz D Katsarava Z Lifting H. The Burden: the Global Campaign against, Migraine remains second among the world's causes of disability, and first among young women: findings from GBD2019. J Headache Pain. (2020) 21:137. 10.1186/s10194-020-01208-0 33267788 PMC7708887

[7] Mayans L Walling A. Acute migraine headache: treatment strategies. Am Fam Phys. (2018) 97:243–51. 29671521

[8] Dodick DW Papademetriou V. Cardiovascular safety of triptans. Editorial. (2004) 24:513–4. 10.1111/j.1468-2982.2003.00714.x 15196291

[9] Chan KY Labruijere S Ramírez Rosas MB de Vries R Garrelds IM Danser AH . A Cranioselectivity of sumatriptan revisited: pronounced contractions to sumatriptan in small human isolated coronary artery. CNS Drugs. (2014) 28:273–8. 10.1007/s40263-013-0136-0 24430784

[10] Edvinsson L. CGRP and migraine: from bench to bedside. Rev Neurol. (2021) 177:785–90. 10.1016/j.neurol.2021.06.003 34275653

[11] Edvinsson L. Role of CGRP in migraine. Handb Exp Pharmacol. (2019) 255:121–30. 10.1007/164_2018_201 30725283

[12] de Vries T Villalón CM MaassenVanDenBrink A. Pharmacological treatment of migraine: CGRP and 5-HT beyond the triptans. Pharmacol Therap. (2020) 211:107528. 10.1016/j.pharmthera.2020.107528 32173558

[13] Tfelt-Hansen P Loder E. The emperor's new gepants: are the effects of the new oral CGRP antagonists clinically meaningful? Headache. (2019) 59:113–7. 10.1111/head.13444 30451300

[14] Tfelt-Hansen P Pascual J Ramadan N Dahlöf C D'Amico D Diener HC . Guidelines for controlled trials of drugs in migraine: third edition. A guide for investigators. Cephalalgia Int J Headache. (2012) 32:6–38. 10.1177/0333102411417901 22384463

[15] Marcus R Goadsby PJ Dodick D Stock D Manos G Fischer TZ. BMS-927711 for the acute treatment of migraine: a double-blind, randomized, placebo controlled, dose-ranging trial. Cephalalgia. (2014) 34:114–25. 10.1177/0333102413500727 23965396

[16] Croop R Goadsby PJ Stock DA Conway CM Forshaw M Stock EG . Efficacy, safety, and tolerability of rimegepant orally disintegrating tablet for the acute treatment of migraine: a randomised, phase 3, double-blind, placebo-controlled trial. Lancet. (2019) 394:737–45. 10.1016/S0140-6736(19)31606-X 31311674

[17] Lipton RB Croop R Stock EG Stock DA Morris BA Frost M . Rimegepant, an oral calcitonin gene-related peptide receptor antagonist, for migraine. N Engl J Med. (2019) 381:142–9. 10.1056/NEJMoa1811090 31291516

[18] Huang T Xu Y Chen Y Bian J Chu Z Zhao S . Efficacy and safety of calcitonin gene-related peptide antagonists in migraine treatment: a meta-analysis. Brain Behav. (2022) 12:e2542. 10.1002/brb3.2542 35261165 PMC9015008

[19] Scott LJ. Rimegepant: first approval. Drugs. (2020) 80:741–6. 10.1007/s40265-020-01301-3 32270407

[20] Johnston KM L'Italien G Popoff E Powell L Croop R Thiry A . Mapping migraine-specific quality of life to health state utilities in patients receiving rimegepant. Adv Ther. (2021) 38:5209–20. 10.1007/s12325-021-01897-2 34455556 PMC8478726

[21] Croop R Lipton RB Kudrow D Stock DA Kamen L Conway CM . Oral rimegepant for preventive treatment of migraine: a phase 2/3, randomised, double-blind, placebo-controlled trial. Lancet. (2021) 397:51–60. 10.1016/S0140-6736(20)32544-7 33338437

[22] Negro A Martelletti P. Rimegepant for the treatment of migraine. Drugs Today. (2020) 56:769–80. 10.1358/dot.2020.56.12.3211624 33332483

[23] Russell FA King R Smillie SJ Kodji X Brain SD. Calcitonin gene-related peptide: physiology and pathophysiology. Physiol Rev. (2014) 94:1099–142. 10.1152/physrev.00034.2013 25287861 PMC4187032

[24] Edvinsson L Warfvinge K. Recognizing the role of CGRP and CGRP receptors in migraine and its treatment. Cephalalgia. (2019) 39:366–73. 10.1177/0333102417736900 29020807

[25] Raddant AC Russo AF. Calcitonin gene-related peptide in migraine: intersection of peripheral inflammation and central modulation. Expert Rev Mol Med. (2011) 13:e36. 10.1017/S1462399411002067 22123247 PMC3383830

[26] Messlinger K Russo AF. Current understanding of trigeminal ganglion structure and function in headache. Cephalalgia. (2019) 39:1661–74. 10.1177/0333102418786261 29989427 PMC7007999

[27] Castrillo A Mendoza A Caballero L Cerdán D Rodríguez MF Guerrero P . Effectiveness of anti-CGRP monoclonal antibodies in the preventive treatment of migraine: a prospective study of 63 patients. Med Clin. (2023) 160:341–6. 10.1016/j.medcle.2022.09.024 36623986

[28] Hoffman V Xue F Ezzy SM Yusuf A Green E Eisele O . Risk of cardiovascular and cerebrovascular events and mortality in patients with migraine receiving prophylactic treatments: an observational cohort study. Cephalalgia. (2019) 39:1544–59. 10.1177/0333102419856630 31195804

[29] Cheng F Ahmed F. Onabotulinumtoxin A for the prophylactic treatment of headaches in adult patients with chronic migraine: a safety evaluation. Expert Opin Drug Saf. (2021) 20:1275–89. 10.1080/14740338.2021.1948531 34187265

[30] Sevivas H Fresco P. Treatment of resistant chronic migraine with anti-CGRP monoclonal antibodies: a systematic review. Eur J Med Res. (2022) 27:86. 10.1186/s40001-022-00716-w 35659086 PMC9167529

